# Pulmonary Alveolar Microlithiasis “Stone Lungs”: A Case of Clinico-Radiological Dissociation

**DOI:** 10.7759/cureus.749

**Published:** 2016-08-24

**Authors:** Andrew Chu, Sameer Shaharyar, Binna Chokshi, Nikhil Bhardwaj

**Affiliations:** 1 Internal Medicine Department GME, Aventura Hospital and Medical Center

**Keywords:** pulmonary alveolar microlithiasis, computed tomography

## Abstract

Pulmonary alveolar microlithiasis (PAM) is a rare infiltrative lung disease characterized by deposition of spherical calcium phosphate microliths called calcospherites within the alveoli. PAM was first described by Friedrich in 1856 and then by Harbitz in 1918. The disease pathogenesis is based on mutations in the SLC34A2 gene that encodes for the Type IIb sodium-phosphate cotransporter. The majority of the patients are diagnosed at an early age, usually between the ages of 20 and 40 years. The hallmark of this disease is a striking dissociation between the radiological findings and the mild clinical symptoms.

We report a case of 35-year-old woman who presented post-motor vehicle accident with back pain and with minimal dyspnea on exertion. The final diagnosis was made after computed tomography and lung biopsy. The present case exhibits the remarkable clinico-radiological dissociation with complete calcification of the lungs on radiographic images with a relatively mild clinical presentation.

## Introduction

Pulmonary alveolar microlithiasis (PAM), or Harbitz’ syndrome, is a rare lung disease characterized by the presence of innumerable small calculi in the alveoli called microliths or calcospherites [1]. Despite the striking radiological appearance, the clinical course is relatively mild and patients remain asymptomatic until the late development of hypoxemia and cor pulmonale. The present report is an example of a “typical” case of PAM with striking radiological features but mild clinical symptoms. 

## Case presentation

**Figure 1:**
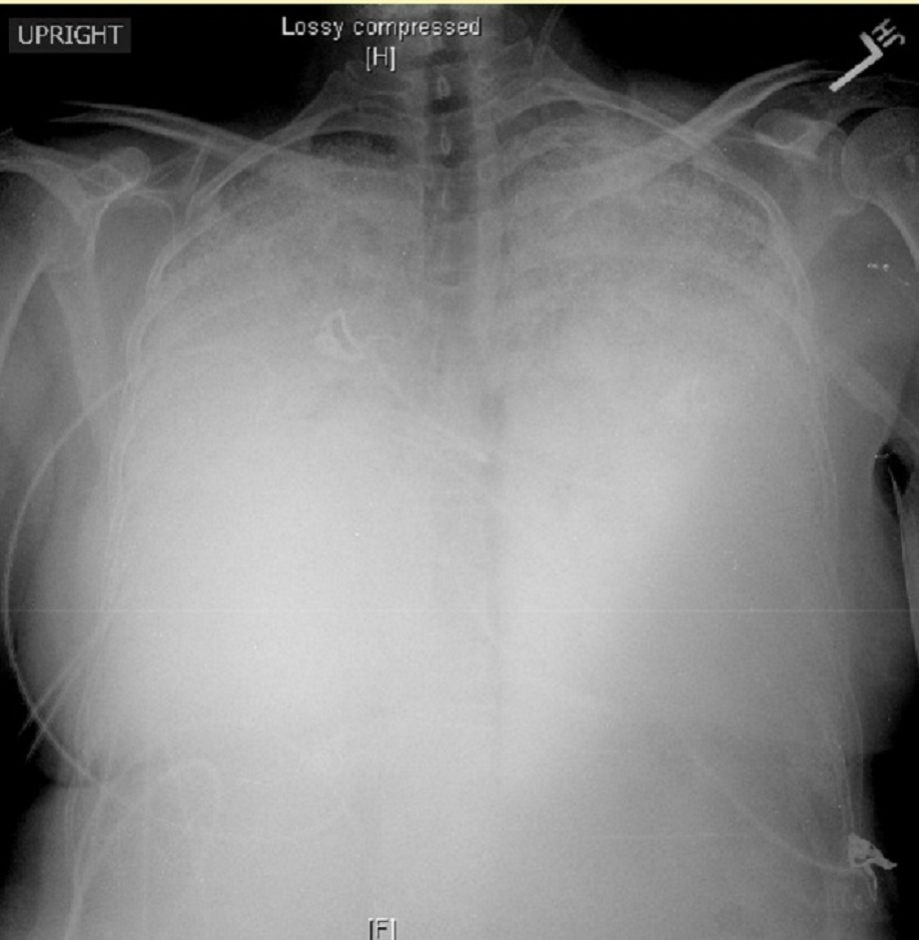
Anteroposterior chest x-ray Dense bilateral pulmonary consolidation with minimal sparing of the lung apices.

The patient had presented as a young 35-year-old female post a motor vehicle accident. She denied past medical and surgical history. She was born in the United States and denied a history of occupational inhalation exposure. She denied smoking tobacco. Upon admission, she was afebrile and hemodynamically stable. Her oxygen saturation was 96.3% on two liters of oxygen delivered via nasal cannula. On physical examination, she had coarse crackles diffusely in bilateral lung fields. Anterior-posterior chest radiograph showed no evidence of rib fractures, but she did have extensive diffuse dense alveolar infiltrates (Figure 1).

**Figure 2:**
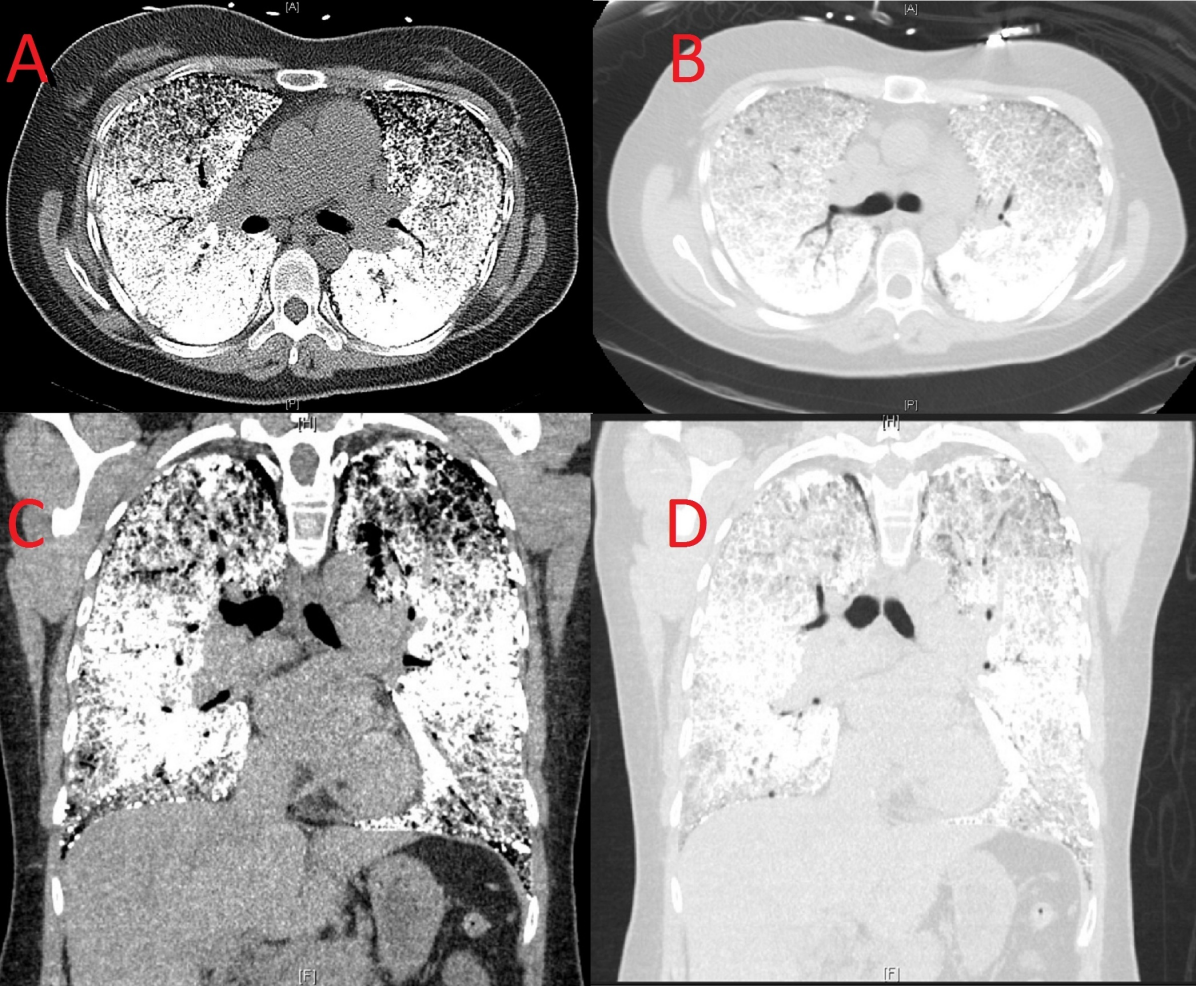
Computed tomography of the chest A: Axial view, intense calcification of the interstitium and pleural serosa. B: Axial view lung window, intense calcification giving a “white lung” appearance. C: Coronal view, intense calcification of the interstitium and pleural serosa sparing the lung apices. D: Coronal view lung window, dense calcification of the lower lung fields with moderate calcification of the apices.

Arterial blood gas showed pH: 7.40, PaCO2: 35.2 mmHg, PaO2: 60.8 mmHg, and HCO3: 22.5 mmol/L on 2 liters of oxygen via nasal cannula. Computed tomography was performed which demonstrated diffuse pulmonary interstitial and alveolar calcification with minimal sparing of the apices (Figure 2).

A transthoracic echocardiogram performed showed systolic and diastolic flattening in the ventricular septum consistent with right ventricular pressure overload. The right ventricular systolic pressure was measured to be 54 mmHg. Biopsy performed on bronchoscopy showed calcified bronchial mucosa. The combination of above-mentioned results and clinical suspicion led to the diagnosis of pulmonary alveolar microlithiasis. The patient did well throughout the hospital stay. As her ambulating oxygen saturation was 88%, she was discharged with home oxygen therapy. Prior to discharge, she was referred to a tertiary care center to be evaluated for possible lung transplantation.

Informed patient consent was obtained for her treatment.

## Discussion

Pulmonary alveolar microlithiasis is a rare autosomal recessive disease with high penetrance. The disease is characterized by extensive radiopaque pulmonary alveolar microliths with only mild clinical features (dyspnea, hypoxia). There have been approximately 1,000 cases reported worldwide since it was first described in 1918 by Harbitz [1]. PAM is present globally, with the largest number of recorded cases in Asia. The nations with the highest number of reported cases per population are Turkey and Italy [2]. The disease has a slight male preponderance (3:2 ratio) and typically presents during the second and third decades of life [2]. However, cases have been reported in neonates up to octogenarians.

PAM is thought to be a genetic disease as a result of a mutation in the SLC34A2 gene, which codes for a sodium phosphate IIb transporter protein. The gene is expressed in epithelial tissues in humans, including mammary glands, the small intestine, kidneys, pancreas, ovaries, liver, testes, placenta, and prostate, in addition to the lungs. With gene dysfunction, alveolar epithelial Type II cells are no longer able to clear phosphorus ions, which leads to the formation of calcium phosphate deposits (microliths) in the extracellular fluid, giving them the characteristic alveolar microlithiasis appearance [3].

Histologically, PAM is characterized by periodic acid–Schiff-positive microliths, typically calcareous concentric lamellae around a central nucleus with an amorphous or granular aspect. These can be differentiated from metastatic and dystrophic calcifications, as the latter are located in the interstitial or vascular compartments [4]. These range from 50 to 1,000 μm in diameter and are predominantly composed of calcium and phosphorus. Although classically involving the lungs, similar calcifications have been noted in PAM patients in extrapulmonary sites, including the male genital tract, kidneys, and prostate.

The characteristic radiologic features have been grouped into four stages. The first phase, known as pre-calcific, denotes the early disease stage without the typical appearance of PAM. The second phase begins to demonstrate the typical radiological appearance of “sandy” or “snowstorm” lungs with diffuse scattered calcific nodules (< 1 mm in size). These tend to be diffusely distributed throughout the lungs, although medial and inferior predilection has been reported. In some cases, these nodules may be larger, resembling berries (~ 2-4 mm). The third phase is characterized by increased number and volume of opacifications, accompanied by some interstitial thickening. At this stage, the outlines of the heart and diaphragm may be obscured by the opacifications. The fourth stage is indicated by an increase in the number and size of calcific deposits leading to intense calcification of the interstitium and pleural serosa. This gives the radiographic typical presentation of a “white lung” appearance, which was the presentation of our patient. Supporting the notion of a chronic process, the first stages are often seen in children, the second in adolescence, and the third and fourth stages in later years of life.

Clinically, PAM has a relatively indolent, albeit non-modifiable, course. Our patient, who presented in Stage 4 of the disease, had relatively mild symptoms in the form of minimal dyspnea, with ambulatory oxygen saturation of 88% [5]. This is in spite of the fact that the disease was chronic and advanced enough to produce cor pulmonale, with evidence of right heart overload. PAM often follows a slow course with years passing prior to clinical symptoms; however, rapidly progressive variants have also been reported in the literature [2].

Differential diagnoses include sarcoidosis, tuberculosis, mycosis, hemosiderosis, pneumoconiosis, and amyloidosis – all diseases with a miliary pattern of dissemination. However, these often have more severe clinical symptoms. Indeed, the relatively mild clinical presentation should prompt consideration of the diagnosis of PAM. It is possible that PAM may be misdiagnosed, especially as tuberculosis in endemic areas, leading to underreporting of the disease.

The diagnosis of PAM can be established with a combination of imaging, tissue sampling, and gene testing. PAM may be diagnosed with reasonable certainty with a combination of the typical appearance on high-resolution computed tomography scan, bronchoalveolar lavage (and/or transbronchial biopsy), and testing for a mutation in the SCL34A2 gene. Therapeutic options remain limited for this disease, with lung transplantation being the only definitive treatment. Calcium chelators, serial bronchopulmonary lavage, and corticosteroids have not been shown to be effective in arresting progression and are considered to have more of a palliative role. Bisphosphonates have been proposed as therapy; however, data on efficacy remains limited. Therefore, lung transplantation remains the only viable treatment option at this time, although there is no prognostic data to determine the indications for the same. Right heart failure or respiratory failure may be considered initial indications for transplant. As yet, there are no reports of recurrence after lung transplant, making it a curative treatment. 

## Conclusions

The present case illustrates the “classic” presentation of a rare genetic disease, pulmonary alveolar microlithiasis, characterized by innumerable alveolar microliths with only mild clinical symptoms but with evidence of right heart failure. A brief overview of the disease is also presented. PAM is a rare disorder with a poorly understood natural history and with lung transplant as the only curative option. The diagnosis should be considered in a patient with severe radiological features, a relatively mild clinical presentation, and especially in the presence of family history or consanguinity. Further study is needed regarding potential therapeutic options for this rare, progressive condition. 
